# Large-scale identification of wheat genes resistant to cereal cyst nematode *Heterodera avenae* using comparative transcriptomic analysis

**DOI:** 10.1186/s12864-015-2037-8

**Published:** 2015-10-16

**Authors:** Ling-An Kong, Du-Qing Wu, Wen-Kun Huang, Huan Peng, Gao-Feng Wang, Jiang-Kuan Cui, Shi-Ming Liu, Zhi-Gang Li, Jun Yang, De-Liang Peng

**Affiliations:** State Key Laboratory for Biology of Plant Diseases and Insect Pests, Institute of Plant Protection, Chinese Academy of Agricultural Sciences, Beijing, 100193 China; State Key Laboratory of Agrobiotechnology and MOA Key Laboratory of Plant Pathology, China Agricultural University, Beijing, 100193 China

**Keywords:** Cereal cyst nematode, *Heterodera avenae*, *Triticum aestivum*, RNA-sequencing, Transcripts filtration, Expression profile, Resistance genes, Defense response

## Abstract

**Background:**

Cereal cyst nematode *Heterodera avenae*, an important soil-borne pathogen in wheat, causes numerous annual yield losses worldwide, and use of resistant cultivars is the best strategy for control. However, target genes are not readily available for breeding resistant cultivars. Therefore, comparative transcriptomic analyses were performed to identify more applicable resistance genes for cultivar breeding.

**Methods:**

The developing nematodes within roots were stained with acid fuchsin solution. Transcriptome assemblies and redundancy filteration were obtained by Trinity, TGI Clustering Tool and BLASTN, respectively. Gene Ontology annotation was yielded by Blast2GO program, and metabolic pathways of transcripts were analyzed by Path_finder. The ROS levels were determined by luminol-chemiluminescence assay. The transcriptional gene expression profiles were obtained by quantitative RT-PCR.

**Results:**

The RNA-sequencing was performed using an incompatible wheat cultivar VP1620 and a compatible control cultivar WEN19 infected with *H. avenae* at 24 h, 3 d and 8 d. Infection assays showed that VP1620 failed to block penetration of *H. avenae* but disturbed the transition of developmental stages, leading to a significant reduction in cyst formation. Two types of expression profiles were established to predict candidate resistance genes after developing a novel strategy to generate clean RNA-seq data by removing the transcripts of *H. avenae* within the raw data before assembly. Using the uncoordinated expression profiles with transcript abundance as a standard, 424 candidate resistance genes were identified, including 302 overlapping genes and 122 VP1620-specific genes. Genes with similar expression patterns were further classified according to the scales of changed transcript abundances, and 182 genes were rescued as supplementary candidate resistance genes. Functional characterizations revealed that diverse defense-related pathways were responsible for wheat resistance against *H. avenae*. Moreover, phospholipase was involved in many defense-related pathways and localized in the connection position. Furthermore, strong bursts of reactive oxygen species (ROS) within VP1620 roots infected with *H. avenae* were induced at 24 h and 3 d, and eight ROS-producing genes were significantly upregulated, including three class III peroxidase and five lipoxygenase genes.

**Conclusions:**

Large-scale identification of wheat resistance genes were processed by comparative transcriptomic analysis. Functional characterization showed that phospholipases associated with ROS production played vital roles in early defense responses to *H. avenae* via involvement in diverse defense-related pathways as a hub switch. This study is the first to investigate the early defense responses of wheat against *H. avenae*, not only provides applicable candidate resistance genes for breeding novel wheat cultivars, but also enables a better understanding of the defense mechanisms of wheat against *H. avenae*.

**Electronic supplementary material:**

The online version of this article (doi:10.1186/s12864-015-2037-8) contains supplementary material, which is available to authorized users.

## Background

Cereal cyst nematode (CCN) *Heterodera avenae* is an important soil-borne wheat pathogen found worldwide that causes significant annual yield losses [1, 2]. Planting and breeding of resistant wheat cultivars are considered as the most economical, environmentally sustainable, and accessible strategies for control of the disease caused by CCN [3]. In past decades, approximately 13 genes with underlying resistance to CCN were identified based on traditional mapping-based cloning strategy, including nine *Cre* genes (1–8, R) and four *Ha* (1–4) genes. Few sources of genetic resistance are documented against CCN in the hexaploid bread wheat *Triticum aestivum.* The dominant resistance gene *Cre1* was characterized in the line Aus 10894/Loros and was extensively used as a source of resistance in commercial breeding programs [4, 5]. Another resistance gene, i.e., *Cre8,* was also derived directly from bread wheat *T. aestivum* [6, 7]. Sources of CCN resistance have been found predominately in wild grasses and relatives of bread wheat, i.e., *Aegilops ventricosa*, *Ae. squarrosa*, and *Ae. triuncialis*, as well as in cultivated rye, i.e., *Secale cereal* [4, 5, 8, 9]. The resistance gene *CreR* was identified from the long arm of 6R within rye [9], whereas the other six *Cre* resistance genes originated from *Aegilops* spp. [3]. In addition, the four resistance genes *Ha1-4* were derived from barley [5, 10]. Moreover, most of these resistance genes conferred resistance to limited or specific pathotypes of CCN [5, 10]. The resistant introgression line H93-8 was generated by transferring the resistance gene *Cre2* from wild grass *Ae.ventricosa* to hexaploid bread wheat and exhibited high resistance to CCN pathotypes [4]. However, applicable resistance genes against *H. avenae* are still limited, and thus, identification of additional genes that confer resistance to CCN are urgently needed.

Isolation and investigation of only a few limited number of resistance genes is time-consuming via the traditional strategy. Recently, transcriptomics has been developed for high-throughput identification of candidate genes of interest and is used to quickly and globally isolate candidate genes against important plant parasitic nematodes, i.e., the soybean cyst nematode *Heterodera glycines* and root knot nematode *Meloidogyne* spp. [11–16]. Many defense-related genes and pathways were identified in incompatible soybean lines during early defense responses to *H. glycines* via comparative transcriptomic analysis [13, 14]. During the resistant reaction of soybean *Glycine max* genotype PI 548402 (Peking) to *H. glycines*, lipoxygenase-9 and lipoxygenase-4 were the most highly induced components. Components of the phenylpropanoid pathway (PAL) (i.e., phenylalanine ammonia lyase, isoflavone reductase, chalcone isomerase, cinnamoyl-CoA reductase and caffeic acid O-methyltransferase) were also remarkably upregulated, which indicated the importance of the jasmonic acid and phenylpropanoid signaling pathways during the early defense response of resistant soybean to *H. glycines* [17]. A microarray analysis was performed using two genetically related soybean sister lines TN02-226 (resistant) and TN02-275 (susceptible) with the race 2 population of *H. glycines*, and the results showed that 42 transcripts increased in the resistant line but decreased in the susceptible line. These genes were associated with metabolism, cell-wall modification, signal transduction, transcription, and defense [13]. During the early defense response of the resistant reaction of *G. max* genotype PI 88788 to *H. glycines* population NL1-RHg/HG-type 7, the jasmonic acid biosynthesis and 13-lipoxygenase pathways were significantly induced in transcript abundance, similar to results from the previous study [13]. The most highly induced pathways contained components of jasmonic acid biosynthesis, flavonoid biosynthesis, 13-lipoxygenase pathway, and phenylpropanoid biosynthesis, which demonstrated that the jasmonic acid defense pathway was involved in the localized resistant reaction of *G. max* [PI 88788] to *H. glycines* [14]. Similarly, global gene expression changes in roots during incompatible and compatible associations with *M. incognita* were also investigated using comparative transcriptomic analysis, and 217 genes were significantly differentially expressed during the time of *M. incognita* infection corresponding to establishment of feeding sites, and 58 genes exhibited differential regulation in resistant roots compared with uninfected roots, including the glycosyltransferase. Functional characterization demonstrated that the glycosyltransferase gene was required for *Mi*-mediated nematode resistance [11]. Until now, no transcriptomic analysis of incompatible defense response to CCN has been reported, and only one report exists on the compatible response of *Ae. variabilis* against CCN [8].

In this study, to apply high-throughput identification of additional candidate genes associated with underlying resistance against CCN, we performed comparative transcriptomic analysis using an incompatible wheat cultivar VP1620 and a compatible wheat cultivar WEN19 inoculated with CCN to achieve the corresponding incompatible (_I) and compatible (_C) reactions at early three time points of 24 h, 3 d, and 8 d. The VP1620 used herein allowed CCN to penetrate and migrate normally within its roots but significantly disturbed the transition of developmental stages, eventually resulting in a dramatic reduction in cyst formation. A novel methodology was generated to establish two types of expression profiles and narrow the scopes of candidate resistance genes. A total of 606 candidate resistance genes were identified, including 484 overlapping genes as well as 122 VP1620-specific genes, and functional characterization analysis indicated that phospholipase was likely to play vital roles against CCN as a hub switch involved in many defense-related pathways.

## Results and discussion

### Resistant wheat VP1620 decreased cyst formation of *H. avenae*

**Fig. 1:**
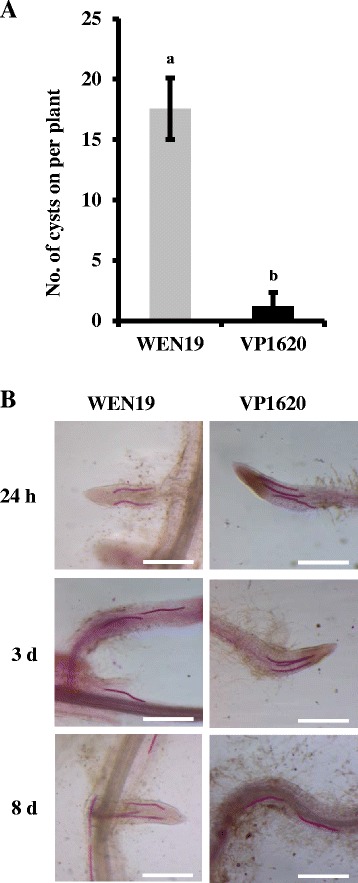
VP1620 significantly decreased cyst formation of *H. avenae:* **a** Number of cysts growing on VP1620. The cyst numbers, which were counted at 90 days post CCN-inoculation on VP1620, were dramatically reduced by approximately eight-fold compared to compatible wheat WEN19. Mean and standard errors were determined using data from three independent replicates. Different letters denote significant differences (*P* value ≤0.05, *n* = 3). **b** Early developmental stages of CCN within VP1620. The CCNs within both VP1620 and WEN19 were examined by staining with acid fuchsin at 24 h, 3 d and 8 d (scale bar = 500 μm)

To globally identify wheat resistance genes against *H. avenae*, an incompatible wheat cultivar VP1620 was used to perform comparative transcriptomic analyses. The number of cysts formed on VP1620 roots was significantly reduced by approximately 8-fold compared to the compatible host WEN19 (Fig. 1a). In this study, the early response of VP1620 against *H. avenae* was characterized by choosing three key time points at 24 h, 3 d, and 8 d, and two main reasons for these choices were taken into consideration. First, these three time points were typical time points that represent the penetration stage (24 h), migration stage (3 d) and feeding site establishment and maintenance (8 d) during the early reaction of parasitism and pathogenesis of *H. avenae*, consistent with the report from a previous study [8]. Second, these three time points allowed *H. avenae* to maintain the post-parasitic Juvenile 2 (post-J2) stage within both compatible wheat WEN19 and incompatible wheat VP1620, and it was both possible and feasible to rule out the impacts of diverse effectors from different developmental stages of CCN in the comparative analysis because the developmental procedures were not synchronous between WEN19 and VP1620 after 8 days post CCN inoculation (Additional file 1: Figure S1; Additional file 2: Table S1). Time-course examinations were assayed at 24 h, 3 d and 8 d after inoculating VP1620 and WEN19 with freshly hatched J2 CCN. The results showed that the numbers of post-J2 CCN within the VP1620 roots were nearly equivalent to those in WEN19 roots at both 24 h and 3d, whereas the number of post-J2 within VP1620 decreased compared with that in WEN19 at 8 d (Fig. 1b; Table 1). Although VP1620 had no significant effect on the penetration efficiency and early developmental process of the post-J2 stage, it had dramatic impacts on the development of the latter J3 and J4 stages. The numbers of total CCN as well as the corresponding latter J3 and J4 stages within VP1620 roots were significantly decreased at 19 d, 25 d and 33 d compared with those of the compatible control WEN19 (Additional file 2: Table S1). Furthermore, the percentages of J3 and J4 were also obviously delayed and decreased vs. those in WEN19 (Additional file 2: Table S1). The VP1620 failed to hamper the penetration and migration of *H. avenae* at the early stage but significantly reduced the total number of CCN in its roots and dramatically disturbed the development of the following J3 and J4 stages. These results strongly demonstrated that the nature of the VP1620 underlying resistance to CCN was anti-development.

**Table 1:** Number of *H. avenae* juveniles in wheat roots of both compatible WEN19 and incompatible VP1620 at early 8-day post-inoculations

Wheat line	24 h	3 d	8 d
WEN19	63.0 ± 10.3^a^	62.3 ± 9.3^a^	65.0 ± 6.4^a^
VP1620	56.3 ± 8.9^a^	55.0 ± 4.8^a^	47.3 ± 5.1^b^

The H. avenae juveniles within wheat roots were stained with 0.01% acid fuchsin solution, and their number was counted with a microscopy. The data were analyzed by using T-test (*P* value ≤0.05, *n*=3). Different letters (a and b) above the numbers denoted significant differences

### A novel strategy was established to assemble and analyse transcriptomic data

**Fig. 2:**
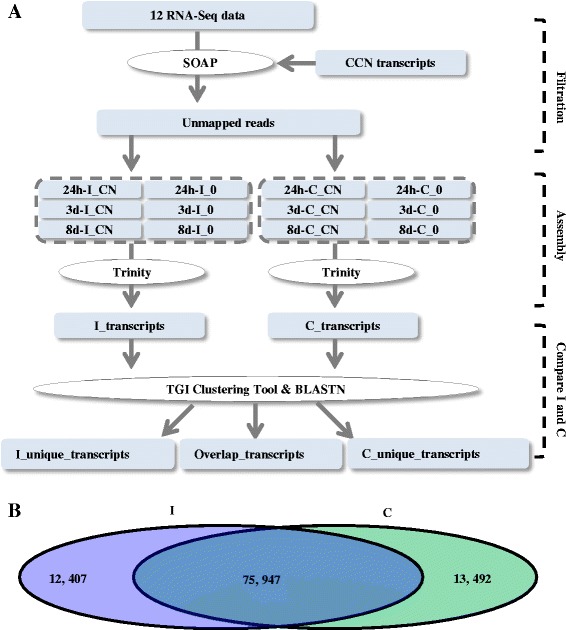
Flowchart of comparative transcriptomic analysis: **a** Transcripts of CCN within raw data were removed before assembly. All six incompatible clean data sets were pooled and assembled with compatible data. **b** A total of 75,947 overlap, 12,407 I_unique and 13,492 C_unique transcripts were produced. Abbreviations: I_CN, incompatible wheat with CCN inoculation; I_0, incompatible wheat without CCN inoculation; C_CN, compatible wheat with CCN inoculation; C_0, compatible wheat without CCN inoculation

For the incompatible wheat VP1620, RNA samples of roots with CCN infections at 24 h, 3 d and 8 d and corresponding samples without CCN inoculation were prepared and sequenced by Illumina Hiseq™ 2000. Because the CCNs are typical sedentary endoparasitic nematodes, they remained within the root tissues until cyst formation [1]. Thus, the root samples of both VP1620 and WEN19 collected at 24 h, 3 d and 8 d after CCN infection also contained an amount of post-J2 CCN within these roots. To rule out their negative effects on the following comparative transcriptomic analysis, the corresponding CCN transcripts were removed according to the SOAP method before assembly (Fig. 2a). The samples of 24 h-I_CN, 3d-I_CN and 8d-I_CN eventually yielded 109,831,800, 106,544,764, and 108,397,110 clean reads after filtration, respectively (Table 2). In contrast, the samples of 24 h-I_0, 3d-I_0 and 8d-I_0 did not require removal of CCN transcripts and generated 110,696,256, 105,034,822 and 110,924,676 clean reads, respectively (Table 2). All six RNA-Seq data sets were pooled and assembled using the Trinity program (Fig. 2a). A total of 137,632 unique transcripts (I_transcripts) were generated with a total length of 96,530,157 nt, a mean length of 701 nt and an N50 of 1257 bp (Table 2). Among the 137,632 transcripts, 112,045 transcripts were larger than 200 nt in length with an approximately percentage of 81.41 %.

**Table 2:** Overview of reads and sequence assembly of both compatible and incompatible transcriptomic data

Sample	Read	Unique transcript	Total Length (nt)	Mean Length (nt)	N50 (nt)	Transcript (>200 nt)
I-24h_0	110,696,256	137,632	96,530,157	701	1,257	112,045
I-24h_CN	109,831,800					
I-3d_0	105,034,822					
I-3d_CN	106,544,764					
I-8d_0	110,924,676					
I-8d_CN	108,397,110					
C-24h_0	107,577,970	142,116	97,125,168	683	1,214	115,305
C-24h_CN	106,514,220					
C-3d_0	109,875,622					
C-3d_CN	106,119,562					
C-8d_0	109,112,640					
C-8d_CN	106,343,260					

Similarly, 24 h-C_CN, 3d-C_CN and 8d-C_CN correspondingly produced 106,514,220, 106,119,562 and 106,343,260 clean reads, whereas 24 h-C_0, 3d-C_0 and 8d-C_0 yielded 107,577,970, 109,875,622 and 109,112,640 reads, respectively (Table 2). The transcriptome of compatible wheat was assembled by pooling all six RNA-Seq data sets using the Trinity program (Fig. 2a). There were 142,116 unique transcripts (C_transcripts) with a total length of 97,125,168 nt, a mean length of 683 nt and an N50 of 1214 bp, respectively (Table 2). A total of 115,305 transcripts had a length greater than 200 nt, and the percentage of transcripts that were greater than 200 nt in length was approximately 81.13 %, which was nearly equivalent to the 81.41 % of the I_transcripts. In addition, the C_transcripts had 4484 more unique transcripts than the I_transcripts, and its total length was 595,011 nt longer than that of the I_transcripts. However, both the mean length and N50 of the C_transcripts were somewhat shorter than those of the I_transcripts (Table 2).

Because incompatible (VP1620) and compatible (WEN19) wheat had different genetic backgrounds, it was necessary to first unify their genetic backgrounds before further comparative analysis. The TGI Clustering Tool and BLANTN were applied to unify their genetic backgrounds (Fig. 2a). A total of 12,407 VP1620-specific (I_unique_transcripts), and 13,492 WEN19-specific (C_unique_transcripts) transcripts were produced (Fig. 2b). Additionally, 75,947 transcripts were shared by both cultivars (Overlap_transcripts). In this manner, we studied the remarkable differences in transcription between the compatible and incompatible responses of wheat against *H. avenae*.

The different responses of compatible or/and incompatible hosts to their plant parasitic nematodes at the early stages were previously investigated [14, 17–19]. However, the transcripts of plant parasitic nematodes were not removed from the raw transcriptomic data, which still contained a certain percentage of endoparasitic nematodes within the roots [8]. In contrast, in this study, we filtered the transcripts of *H. avenae* from the detected transcriptomics prior to comparative analysis (Fig. 2). This process represents a novel modification that yields precise and clean transcriptomic data and ensures the credibility and accuracy of the transcriptomic data for further analysis.

### Upregulated genes in VP1620 induced by *H. avenae* were identified

**Fig. 3:**
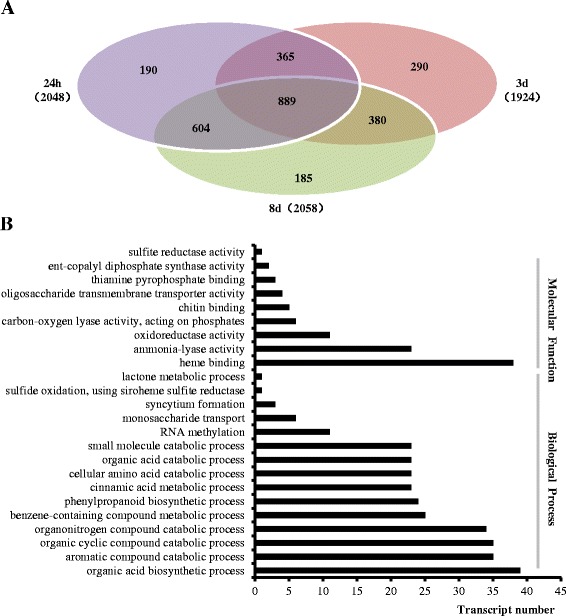
Identification and functional classification of upregulated genes of VP1620 after CCN infestation: **a** Venn diagram showing the numbers of genes upregulated at 24 h, 3 d and 8 d, among which the genes with remarkable induction in at least one of the three time points were considered as significantly upregulated genes. **b** A total of 889 significantly upregulated genes were functionally classified into “Molecular Function” and “Biological Process” based on GO annotation

To identify upregulated genes of VP1620 as an early defense response of wheat to *H. avenae*, a comparative transcriptomic analysis of 24 h-I_CN, 3d-I_CN and 8d-I_CN was performed. Groups of 2048, 1924, and 2058 upregulated genes were identified at 24 h, 3 d and 8 d, respectively (Fig. 3a). Among these genes, 889 genes were simultaneously overlapping among 24 h-I_CN, 3d-I_CN and 8d-I_CN and significantly upregulated in at least one of the three time points (Fig. 3a). A total of 1349 genes were common between any two time-point pairs, including 365 genes between 24 h and 3 d, 604 genes between 24 h and 8 d, and 380 genes between 3 d and 8 d. The number of unique genes at 24 h, 3 d and 8 d was 190, 290 and 185, respectively (Fig. 3a).

To explore their “Molecular Function” and “Biological Process”, 889 genes were categorized into subgroups based on GO analysis, and approximately 50 % genes contained GO information. For “Molecular Function”, the “heme binding” subgroup had the largest number with 38 genes (Fig. 3b), a result similar to that of a previous study in which Dap1p (damage resistance protein 1) bound to heme and stabilized the cytochrome P450 protein Erg11p/Cyp51p to result in antifungal resistance [20]. The Dap1p-cytochrome P450 protein pathway is broadly conserved in eukaryotic species [20, 21]. The following two large subcategories were “ammonia-lyase activity” and “oxidoreductase activity” with 23 and 11 genes, respectively (Fig. 3b). The percentage of the top three GO subdivisions accounted for approximately 77.42 %. Defense-related secondary metabolite phenylalanine ammonia-lyase (PAL) activity was significantly induced in response to *H. avenae*, which was consistent with a previous study in which PAL was remarkably induced in early defense reactions of resistant soybean to *H. glycines* [13]. The PAL activities were also obviously enhanced within the defense responses to pathogens and aphids [22, 23]. Our results, combined with those of the previous studies, demonstrated that PAL may play vital and general roles against diverse pests. Oxidoreductase activity was dramatically enhanced in response to *H. avenae*, which was consistent with the responses of soybean cyst nematode and pine wood nematode [24, 25]. An oxidoreductase, i.e., the 2OG-Fe (II) oxygenase family protein with high abundant transcripts, was identified from resistant soybean induced by soybean cyst nematode [24]. Moreover, the “oxidoreductase activity” subcategory was also present with a high percentage in resistant pine infected by pine wood nematode [25]. The high percentage of oxidoreductase strongly suggested that oxidoreductase was likely to play conserved roles in defense responses to diverse plant parasitic nematodes.

The “Biological Process” is primarily composed of diverse catabolic, biosynthetic and metabolic processes. There were 11 significantly enriched subcategories with more than 10 genes in each subcategory (Fig. 3b). A total of 295 upregulated genes were included in these 11 subcategories. It is clear that most of the subcategories were strongly involved in catabolic processes (Fig. 3b), indicating that catabolic processes played important roles in resistance against *H. avenae,* likely due to sufficient yield and essential precursors for biosynthesis of antimicrobial components. The functional group with the highest number (39 genes) was “organic acid biosynthetic process” (Fig. 3b). The subcategory “benzene-containing compound metabolic process”, which included 25 genes, was also significantly enriched (Fig. 3b), consistent with identification of a similar subcategory within an incompatible reaction of Arabidopsis to *Pseudomonas syringae* [26]. The phenylpropanoid pathway is considered closely related to plant defense against nematode infection [13, 14, 27]. Identification of the subcategory “phenylpropanoid metabolic process” with 24 genes (Fig. 3b) was consistent with a previous study in which the PAL gene was specifically upregulated in the resistant tomato induced by potato cyst nematode *Globodera rostochiensis* [27], suggesting key roles for PAL-mediated metabolism in *H. avenae* resistance [27]. It is well known that lignin biosynthesis plays vital roles in the defense response [28]. The subcategory “cinnamic acid metabolic process” contained 25 genes, demonstrating that a cinnamic acid metabolic process was likely to participate in *H. avenae* resistance resulting from stiffening of the cell wall by enhanced lignin biosynthesis.

Monosaccharide transport and syncytium formation are two essential processes for feeding site establishment and maintenance, which serve as the sole nutrient sink in which plant parasitic nematodes develop and parasitize [1]. Two subcategories of “syncytium formation” and “monosaccharide transport” with four and six genes, respectively, were also identified as induced (Fig. 3b), illustrating that VP1620 had a potential ability to feed *H. avenae*, although it was eventually proven to be highly resistant to CCN, which was consistent with the results mentioned above that VP1620 allowed *H. avenae* to develop into a few cysts (Fig. 1a).

Among those common 889 genes, 18 genes were dramatically upregulated simultaneously among the three time points (Additional file 3: Figure S2). Most of the 18 vital resistance genes were defense-related components, including one pathogenesis-related protein, one elongation factor-1 α-like protein, one probable mediator of RNA polymerase II transcription subunit, three cell wall-associated hydrolase genes associated with innate immune detection, phytoalexin biosynthesis-related protein premnaspirodiene oxygenase-like, two potent antioxidant protein curcuminoid synthase-like, and lignin biosynthesis involving proteins cationic peroxidase SPC4 and cinnamoyl-CoA reductase 1 (Additional file 4: Table S2).

### Candidate resistance genes were identified from overlapping genes

**Fig. 4:**
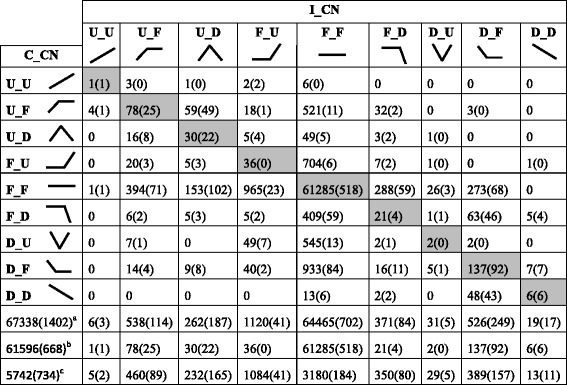
Expression profiles of overlapping genes: Cross-table representation of the expression profiles of overlapping genes within I_CN and C_CN. Numbers outside brackets represent the numbers of all considered genes except growth- and development-related genes, and the numbers within brackets represent numbers of significantly upregulated genes induced by CCN infestation. Each cluster combination within the dataset is indicated in each square. The top and left squares show trend lines for changes in expression pattern across three time points. Abbreviation: U, up-forward trend; D, down-forward trend; F, unchanged trend. ^a^ Total numbers of genes displaying expression profiles of each I_CN combined with nine C_CN; ^b^ Total numbers of genes displaying similar expression profiles on the diagonal line; ^c^ Total numbers of genes displaying uncoordinated expression profiles outside the diagonal line

To globally identify resistance genes, expression profiles of all overlapping genes within VP1620 infected by *H. avenae* (I_CN) were compared with those of the compatible version (C_CN). The expression trends of both I_CN and C_CN displayed nine types at three time points covering the former stage of 24h_vs_3d (from 24 h to 3 d) and the latter stage of 3d_vs_8d (from 3 d to 8 d). Because the time intervals spanned a long period (seven days), a great number of growth and development-related genes were quickly and highly induced and could be detected by comparing C_0 and I_0. To rule out the interfering effects of these genes on identification of resistance genes, we removed these genes before comparing the expression profiles. A total of 67,338 overlapping genes were generated after filtration (Fig. 4). Among the 67,338 overlapping genes, 61,596 genes shared similar expression profiles, as shown outside the brackets on the diagonal line (Fig. 4), which suggested that these genes were likely independent of CCN resistance. However, 5742 genes had uncoordinated expression patterns, as shown outside the brackets beyond the diagonal line (Fig. 4), which indicates that these genes were likely associated with CCN resistance.

To narrow the scopes of the genes that exhibit diverse expression profiles, the genes with expression levels that were obviously simultaneously unchanged among the three time points I_CN compared with VP1620 infected by water (I_0) were wiped off, whereas the genes induced at one of the three time points in I_CN compared with I_0 were retained for further analysis. A total of 1402 genes were ultimately yielded after this procedure (Fig. 4). Among the 1402 genes, 668 genes were found to share similar expression profiles, as shown within the brackets on the diagonal line, whereas 734 genes exhibited uncoordinated expression patterns between I_CN and C_CN, as shown within the brackets beyond the diagonal line (Fig. 4). Obviously, these 734 genes are the most potential to be associated with the wheat resistance to CCN.

It is well known that most of the plant resistance genes were highly induced by diverse pathogens, and thus, in this study, the significantly upregulated genes either in 24h_vs_3d or in 3d_vs_8d were viewed and treated as candidate resistance genes. Among those 734 potential genes described above, a total of 302 genes were dramatically induced with uncoordinated expression profiles between I_CN and C_CN (Fig. 4) and taken as the candidate resistance genes. Among these 302 candidate resistance genes, only 2 genes exhibited continuously enhanced expression trends (U_U) in both the 24h_vs_3d and 3d_vs_8d stages, whereas 254 genes displayed apparently increased expression trends in the 24h_vs_3d stages, including 89 genes with an unchanged expression trend (U_F) and 165 genes with sharply decreased expression tendency (U_D) in the 3d_vs_8d stages; however, 46 genes exhibited dramatically increased expression trends in the 3d_vs_8d stage, including 41 genes that maintained an unaltered expression trend (F_U) and 5 genes that showed obviously decreased expression tendencies (D_U) 24h_vs_3d stages (Fig. 4). Clearly, the number of genes that exhibited significantly increasing expression trends in the 24h_vs_3d stages were increased by approximately 5.5-fold compared to those in the 3d_vs_8d stages, indicating that dramatic transcriptional changes in the early stage were likely to determine the following resistant characteristics.

### Phospholipase functions as a central regulator in resistance against *H. avenae*

**Fig. 5:**
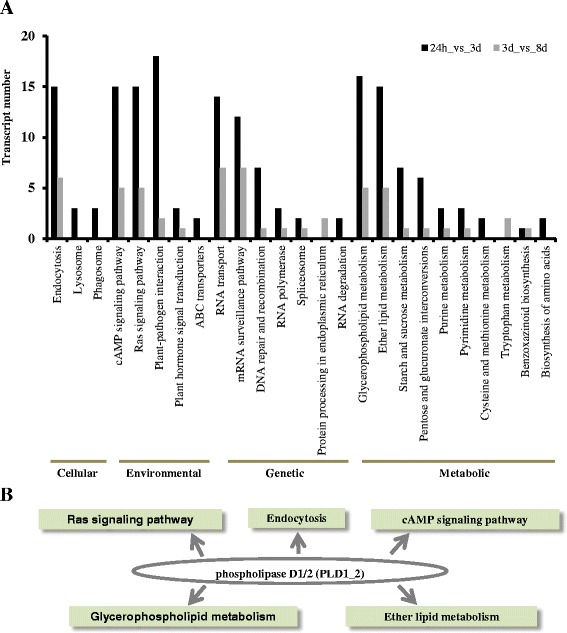
KEGG enrichment analysis of genes exhibiting significantly up-forward trends within I_CN: **a** KEGG enrichment tests were analyzed with the genes exhibiting dramatically up-forward expression patterns in both the 24 h_vs_3d and 3d_vs_8d stages. Cellular, environmental, genetic and metabolic pathways were obviously enriched. **b** Phospholipase was a central regulator during early response to CCN. Phospholipase D1/2 (PLD1_2) was a key component among five pathways and as a hub switch

To obtain an overview of the processes altered during the early stages of VP1620 in defense as a response to *H. avenae* infestation, the 302 candidate resistance genes identified above were functionally classified by KEGG analysis. A total of 224 genes out of 302 genes, including 169 genes that exhibited increasing expression trends in the 24h_vs_3d stage and 55 genes that displayed increasing expression trends in the 3d_vs_8d stage, were categorized into 50 pathways (Additional file 5: Table S3), among which 25 pathways had at least two genes (Fig. 5a). Six pathways, i.e., “Lysosome”, “Phagosome”, “ABC transport”, “RNA degradation”, “Cysteine and methionine metabolism” and “Biosynthesis of amino acid”, contained KEGG information only in the 24h_vs_3d stage with a total number of 14 genes (Fig. 5a). In contrast, the two pathways of “Protein processing in endoplasmic” and “Tryptophan metabolism” contained KEGG information only in the 3d_vs_8d stage with a total number of four genes (Fig. 5a). The “Glycerophospholipid metabolism” pathway was enriched with the largest number of members (21 genes), among which were 16 genes in the 24h_vs_3d stage and five genes in the 3d_vs_8d stage. A total of 20 genes were enriched in “Ether lipid metabolism”, including 15 and five genes in the 24h_vs_3d and 3d_vs_8d stages, respectively. Another eight enriched KEGG-pathways contained members beyond 10 genes (Fig. 5a). Our results indicated that the number of genes dramatically enhanced during the former stage (24h_3d) was significantly larger than that in the latter stage (3d_8d), which supports the hypothesis that early reaction of the incompatible defense would determine the subsequent and even the final phenotypes or resistant characteristics.

**Fig. 6:**
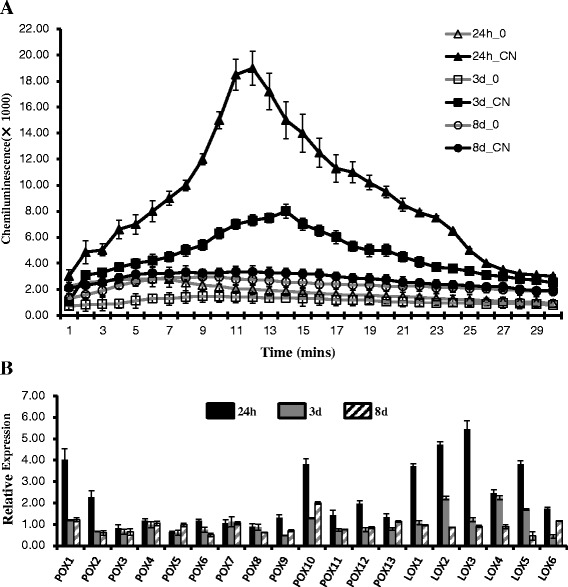
Production of ROS and induction of ROS-producing genes within VP1620 respond to *H. avenae* infestation: **a** ROS contents within infected and uninfected roots of VP1620 at three time points after CCN infection. The ROS content was determined by a luminol-chemiluminescence assay (_0, no CCN inoculation; _CN, CCN inoculated lines; error bars represent the SE (*n* = 3)). **b** Identification of ROS-producing genes in VP1620 significantly induced after *H. avenae* infestation and relative expression detection assayed by qRT-PCR. The expression level of each ROS-producing gene in I_0 was arbitrarily set to 1. Abbreviations: POX, Class III Peroxidase; LOX, Lipoxygenase. Mean and standard errors were determined using data from three independent replicates

Endocytosis has been demonstrated as involved in plant immunity through many protein–protein interactions [29, 30]. The “Endocytosis” pathway was enriched with 15 genes in the 24h_vs_3d stage and six genes in the 3d_vs_8d stage (Fig. 5a), indicating that endocytosis is also likely involved in the defense response to CCN infestation. It has been reported that lysosomes are involved in plant defense by participation in autophagy leading and programmed cell death contributing to the defense response [31]. Unlike “Endocytosis”, both “Lysosome” and “Phagosome” had only three genes within the 24h_vs_3d stage (Fig. 5a), suggesting that lysosomes were likely to function during the early defense reaction to CCN attack.

Interestingly, in this study, two genes involved in the “benzoxazinoid biosynthesis” pathway, DIMBOA (2, 4-dihydroxy-7-methoxy-1, 4-benzoxazin-3-one) which is a naturally occurring hydroxamic acid, and benzoxazinoid, were identified (Fig. 5a). DIMBOA is a powerful antibiotic present in maize and related grasses, particularly wheat, and serves as a natural defense against a wide range of pests, including insects, pathogenic fungi and bacteria. The identification of significantly upregulated expression genes involved in the “benzoxazinoid biosynthesis” pathway showed that DIMBOA was also likely to play an important role in defense against plant parasitic nematodes.

The RNA metabolism was likely involved in resistance to *H. avenae*. The RNA methylation probably plays vital roles in resistance to *H. avenae*, similar to a new resistance mechanism of resistance against aminoglycosides among gram-negative pathogens according to methylation of ribosomal RNA [32]. In genetic pathways, the “RNA transport”, “mRNA surveillance pathway” “DNA repair and recombination” pathways contained 14, 12 and 7 genes, respectively, in the former 24h_vs_3d stage, and 7, 7 and 1 gene, respectively, in the latter 3d_vs_8d stage (Fig. 5a). In this study, the “RNA methylation” subgroup included 11 genes that were also identified (Fig. 3b), indicating that RNA methylation likely played a vital role in resistance to *H. avenae*. It has been reported that a candidate resistance gene of maize against *Aspergillus flavus* was a component of the nuclear pore complexes (NPCs) in the nuclear envelope, and NPCs were involved in RNA transport [33]. The RNA transport pathways are composed of various protein complexes that regulate gene expression and nucleocytoplasmic trafficking. The nucleocytoplasmic trafficking pathways are fundamental for normal cell functions as well as plant defense responses [34]. Previous studies demonstrated that RNA transport pathway genes played direct roles in plant defense systems [33, 35]. The mRNA surveillance pathway, also known as nonsense-mediated mRNA decay (NMD), represents a splicing- and translation-dependent pathway of RNA degradation, which limits the expression of transcripts bearing premature translation termination codons [36–38]. In addition, the DNA repair and recombination pathway was also enriched (Fig. 5a). The DNA repair and recombination pathways have many functions in different species, i.e., maintenance of genomic integrity, chromosome segregation and recombination, and immune system development. The *Arabidopsis* RAR genes include homologues of many DNA repair genes that are defective in different human diseases [39–41]. To the best of our knowledge, this paper is the first report that RNA and DNA metabolism is likely involved in plant immunity against plant parasitic nematodes.

The KEGG pathway enrichment tests showed that the cAMP signaling pathway, Ras signaling pathway, glycerophospholipid metabolism, and ether lipid metabolism contained large numbers of gene members (Fig. 5a). The cAMP signaling pathway involves the stress response through accumulation of phytoalexin and ethylene production by regulating Ca^2+^ or K^+^ flux and thereby initiates Ca^2+^ and protein kinase signaling cascades [42]. Ras are signaling proteins known to transmit extracellular signals in yeast and animals and have a crucial role in cellular signaling in animals and various lower eukaryotes [43, 44]. Recently, a plant intracellular Ras-group LRR family in Arabidopsis was identified and proven to function in development [44]. Members of the Ras superfamily share several common structural features, including four guanine nucleotide binding domains and an effector binding domain, and potentially function in intracellular defense signal transduction via protein-protein interactions mediated by these typical domains [45]. Glycerophospholipid serves as a structural component of cell membranes, which provides a robust permeability barrier and a first line of defense against different stresses; additionally, the glycerophospholipid pathway was previously revealed as critical for programmed cell death, which is involved in the defense response to attacks from diverse pathogens [46, 47]. Ether lipid metabolism was likely to associate with the lipoxygenase (LOX) pathway to generate antimicrobial compounds such as the divinyl ether fatty acids, leading to defense responses [48]. Furthermore, our results also demonstrated that several vital LOX genes were also significantly upregulated and induced by *H. avenae* infestation (Fig. 6).

**Fig. 7:**
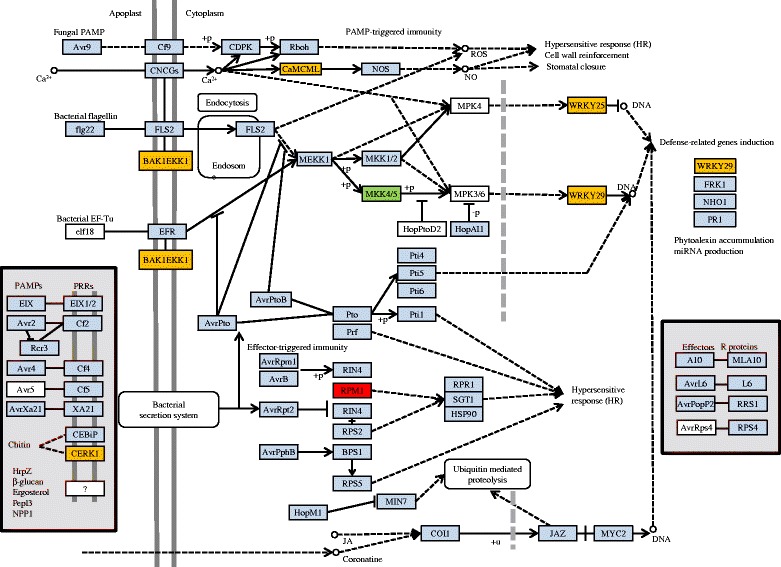
Plant-pathogen interactions (PPI) pathway: Seven genes were enriched in the PPI pathway, five genes in orange were significantly upregulated in the 24 h_vs_3d stage, and one gene in green was dramatically upregulated in the 3d_vs_8d stage, whereas only one gene in red was constantly upregulated in both stages. The PPI pathway was modified from the KEGG website

Phospholipase D (PLD) has been shown to be involved in both abiotic and biotic stress signaling [49, 50]. Interestingly, in this study, phospholipase D1/2 coding genes (PLD) were found in many of the vital and large KEGG pathways mentioned previously, i.e., “Endocytosis”, “cAMP signaling pathway”, “Ras signaling pathway”, “Glycerophospholipid metabolism”, and “Ether lipid metabolism” (Fig. 5b). Moreover, PLD localized in the connection position and linked these pathways to form a complex network (Fig. 5b). This finding strongly suggested that phospholipase was likely to be as a central regulator in resistance to cereal cyst nematode *H. avenae*.

It has been reported that phospholipase positively mediated plant defense response through its role in resistance signaling associated with reactive oxygen species (ROS) [49–51]. To characterize the role of ROS in defense response of I_CN at the early stages, we assayed the ROS contents within VP1620 infected by CCN at 24 h, 3 d and 8 d. The results showed two ROS bursts, including a strong ROS burst at 24 h and an obviously lower ROS burst at 3 d (Fig. 6a). However, no dramatic change of ROS content was detected at 8 d (Fig. 6a). Moreover, 19 ROS-producing genes, including 13 Class III peroxidase (POX) genes and six lipoxygenase (LOX) genes, were identified from overlapping genes between _I and _C (Additional file 6: Table S4). The qRT-PCR results showed that three of 13 POXs were significantly upregulated at 24 h after CCN infestation, and their changes in relative expression were more than two-fold; however, these three genes had no obvious induction of transcript abundance at either 3 d or 8 d (Fig. 6b). Compared with a low percentage (23 %) of POX genes responsible for ROS production, five of the six (83 %) LOX genes identified herein were dramatically upregulated at 24 h after CCN inoculation. In addition, LOX2 and LOX4 were also significantly induced by CCN at 3 d (Fig. 6b). These results showed that significant upregulation of POX combined with LOX were responsible for the former strong ROS production at 24 h, and only the dramatic induction of LOXs contributed to the latter ROS burst. As a whole, lipoxygenase was primarily responsible for ROS bursts in VP1620 after CCN infestation. Our results further supported the roles of phospholipase in wheat defense responses to *H. avenae*.

### Plant-pathogen interaction was significantly enriched

In this study, “plant-pathogen interaction (PPI)” was enriched with the largest numbers (20 genes), including 18 genes in the 24h_vs_3d stage and two genes in the 3d_vs_8d stage, respectively (Fig. 5a). Seven key defense-related genes were dramatically upregulated (Fig. 7). Among these seven genes, five genes were found to be significantly upregulated in the 24h_vs_3d stage, including two WRKY transcription factor genes (WRKY25, WRKY29), one MAPK cascade gene (BAK1), one Ca^2+^ calmodulin-like protein (CaMC/ML) gene, and one CERK1 gene; however, only one MKK4/5 gene (two redundant MAPKKs) showed dramatically enhanced expression in the 3d_vs_8d stage. Furthermore, one gene RPM1 was remarkably and continuously upregulated in both 24h_vs_3d and 3d_vs_8d stages (Fig. 7).

Chitin is a main component of the cell wall of fungal pathogens [52, 53], the exoskeletal cuticle and gut lining of insects [54, 55], and the egg shell of the nematode [56, 57]. Previous studies indicated that CEBiP (chitin elicitor-binding protein) and its partner LysM-RLK (receptor-like kinase) chitin elicitor receptor kinase1 (CERK1) are involved in plant immunity by recognizing the chitin oligosaccharides of the cell wall of fungal pathogens through extracellular LysM domain [58, 59]. In this study, ten genes were identified with LysM domain, and only one gene CERK1, which not only had orthologs to both AtCERK1 and OsCERK1 but also had an extra tyrosine kinase domain, was remarkably enhanced in the incompatible response against CCN infestation (both 3dpi and 8dpi) (Fig. 7), while the other nine genes had no orthologs to AtCERK1 or OsCERK1. One of these nine genes was obviously down-regulated (3dpi), while the other eight genes had no significant changes in their expression level. These results indicated CERK1 played potential roles in defense responses to CCN. Recently, it was reported that chitin-binding protein was also associated with plant defense against the insect Hessian fly [60]. Our finding of CERK1 gene associated with defense response to *H. avenae*, combined with previous studies on similar functions with fungal pathogens and insect, indicated that CERK1 activated defense-related genes by recognizing chitin not only from fungal pathogens but also from insect and plant parasitic nematode, which was treated as a supplemental view for current perspectives. It has been shown that the receptor-like kinase (RLK) pattern recognition receptor (PRR) protein CERK1 perceives the fungal pathogen-activated molecular patterns (PAMPs) chitin to trigger a PAMP-triggered immunity (PTI) response [61]. The PTI signaling requires its dimerization with the receptor-like protein CEBiP1 through direct binding with chitin [61, 62]. In this study, it is likely that putative CERK1 was also involved in PTI response of incompatible wheat to CCN, possibly through recognition of the chitin component of CCN. Plant CaM/CML induced downstream nitric oxide (NO) synthesis as an intermediary sites in a pathogen perception signaling cascade, eventually leading to innate plant immune responses [63]. Plant MAPKs (mitogen-activated protein kinases) cascades play pivotal roles in regulating plant development and signaling responses to a variety of stress stimuli. Activation of MAPKs is performed by their upstream kinases, i.e., MAPK kinases (MAPKKs), and MKK4/MKK5 as a type of MAPKKs acted upstream of MPK3/6 in regulating plant development and defense responses by regulating jasmonic acid (JA) signal transduction [62]. In this study, pathogenesis-related (PR) genes orthologous to PR4 and PR10, which involves the defense meditate by JA pathways [64, 65], were significantly up-regulated at different times. However, most of the other PR genes were not obviously changed in their expression level in the event of CCN attack. These results indicated that JA pathway may play a pivotal role in wheat to CCN similar to that of rice defense against root knot nematodes [64].

The BAK1 (brassinosteroid insensitive 1 associated kinase 1) is a positive regulator of the brassinosteroid (BR) hormonal signaling pathway [61]. The BAK1 was significantly up-regulated (Fig. 7), while both FLS2 (FLAGELLIN SENSITIVE2) and EFR (elongation factor Tu (EF-Tu) receptor) were not obviously changed. FLS2 and EFR are two vital receptors to recognize the specific components of bacterial pathogens to activate PAMP-triggered immunity [66, 67]. So, the BAK1 is a ligand-independent co-receptor of RLKs such as FLS2 and EFR and acts as a central regulator of PTI triggered by diverse PAMPs [61]. The enhanced expression of both MKK4/MKK5 and BAK1 in this study resulted in PTI of incompatible wheat to CCN, likely according to association with the plant hormone network. Many WRKY genes were proven as involved in innate plant immune responses and showed increased expression in response to diverse pathogens, i.e., fungal and bacterial pathogens [61, 62], soybean cyst nematode [68] and root knot nematode [69], as well as cereal cyst nematode *H. avenae* in this study. The RPM1 encodes a peripheral membrane protein with LZ-NBS-LRR domains and confers resistance to specific *Pseudomonas syringae* strains [70]. In addition, the locus of leaf rust resistance gene Lr10 was similar to the *Arabidopsis* RPM1 locus [71]. Resistant complex components of RAR1, SGT1 and HSP90 played vital roles in defense response to diverse pests. RAR1 was originally isolated from barley *Hordeum vulgare* and is present in eukaryotes except for yeast *Saccharomyces cerevisiae*. RAR1 was required for a subset of R-gene-mediated resistance responses in monocot and dicot plant species [72, 73]. SGT1 interacting with RAR1 also contributed to R-gene-mediated resistance [74]. The complex of both RAR1 and SGT1 interacting with chaperon HSP90 functioned in various signal transduction networks [74, 75]. However, the complex of HSP90 and SGT1 (except for RAR1) was required for *Mi*-1-mediated aphid and nematode resistance [72], providing further evidence for common components in early resistance defense signaling against diverse pathogens and pests. In this study, the PRM1 was significantly upregulated in both the 24h_3d and 3d_8d stages, strongly suggesting that PRM1 likely served as a constitutive defense gene involved in the resistance to *H. avenae*. Whether the total or partial complex components of SGT1, RAR1 and HSP90 are required for resistance to *H. avenae* must be further investigated.

### Additional rescued candidate resistance genes were identified

For genes that exhibit similar expression profiles, their scales of altered expression levels could remarkably differ between I_CN and C_CN. To identify additional resistance genes from the genes that exhibited similar expression profiles, the expression profiles were analyzed using the scales of altered expression levels. Among the 668 genes exhibiting similar expression profiles (Fig. 4), 182 genes were rescued and isolated with uncoordinated expression patterns, and 486 genes were identified with similar expression profiles using the scales of altered expression levels as a standard (Additional file 7: Table S5). Among the overlapping genes, most of the candidate resistance genes were identified from uncoordinated expression patterns according to transcript abundance as a standard. Additional resistance genes were subsequently rescued from similar expression profiles with the scales of transcriptional changes as a standard. In this study, we combined both strategies to develop a further comprehensive methodology to identify additional candidate resistance genes not only from uncoordinated expression profiles as the main section but also from similar expression patterns as a supplementary component.

The KEGG pathway analysis revealed that “phenylalanine metabolism”, “phenylpropanoid biosynthesis” and “diterpenoid biosynthesis” involving phytoalexin biosynthesis were enriched with four or five genes, respectively, and “RNA transport” and “mRNA surveillance pathway” were also dramatically enriched with 14 and six genes, respectively. In contrast, only one member of the LRR receptor-like serine/threonine-protein kinase FLS2 was identified with typical characteristics of a resistance gene in “plant-pathogen interaction pathway” (Additional file 8: Table S6). Moreover, defense-related pathways “Endocytosis”, “cAMP signaling pathway”, “Ras signaling pathway”, “Glycerophospholipid metabolism”, and “Ether lipid metabolism” were also significantly enriched, and these pathways were cross-linked to form an extraordinary network by the hub switch phospholipase D1/2, similar to findings mentioned previously (Fig. 7).

### VP1620-specific resistance genes were also identified

To identify VP1620-specific defense-related genes as the candidate resistant genes, incompatible unique (I_unique) transcripts were classified using expression profiles with transcript abundance as a standard. A total of 150 genes were significantly upregulated at one of the three time points in I_CN compared with I_0 (Additional file 9: Figure S3). Therefore, these 122 genes can be taken as the candidate resistance genes. Among these genes, 122 genes had uncoordinated expression profiles, as shown beyond the diagonal line, and 28 genes shared similar expression profiles, as shown on the diagonal line (Additional file 9: Figure S3). The number of genes exhibiting uncoordinated expression profiles was approximately four times that of similar expression profiles. It is clear that 88 % of the 150 genes (132 genes) had an F_F expression patterns in I_0 vs. those that had diverse expression profiles in I_CN. For these 132 genes, except for 28 genes that showed similar expression trends between I_CN and I_0, 63 genes out of the left 104 genes displaying uncoordinated expression profiles were significantly upregulated in at least one of the three time points (Additional file 9: Figure S3). A total of 17 vital VP1620-specific defense related genes were identified (Table 3). Among these 17 genes, seven protein kinase coding genes, including three proline-rich receptor-like protein kinases, one G-type lectin S-receptor-like serine/threonine-protein kinase, one protein-kinase-domain-containing protein, one cysteine-rich receptor-like protein kinase and one probable LRR receptor-like serine/threonine-protein kinase (except for one proline-rich receptor-like protein kinase) exhibited similar expression profiles U_D in I_CN (Table 3). Among the three transcription factor genes, one MADS-box transcription factor displayed a U_D expression pattern, whereas the other two ethylene-responsive transcription factor ABI4 exhibited U_F in I_CN (Table 3). Among the seven resistance genes, two leucine-rich repeat (LRR) genes and one resistance RGA2 gene exhibited U_D expression, and two hydroxyproline-rich glycoprotein genes displayed the corresponding F_U and U_D expression profiles in I_CN (Table 3). Moreover, the results of KEGG pathway enrichment analysis also indicated that phospholipase in partially VP620-specific components was involved in many defense-related pathways similar to those mentioned previously.

**Table 3:** Representatives of VP1620-specific defense-related transcripts

Gene ID	I_CN	I_0	Annotation
Kinase			
Unigene43155_xckb	D_U	F_F	Proline-rich receptor-like protein kinase PERK2
Unigene66089_xckb	U_D	F_F	G-type lectin S-receptor-like serine/threonine-protein kinase SD1-13
Unigene13595_xckb	U_D	F_F	Protein kinase domain containing protein
CL3347.Contig1_xckb	U_D	F_F	Cysteine-rich receptor-like protein kinase 8
Unigene60834_xckb	U_D	F_F	Probable LRR receptor-like serine/threonine-protein kinase At4g29180
Unigene68690_xckb	U_D	F_F	Proline-rich receptor-like protein kinase PERK10
Unigene35121_xckb	U_D	F_F	Proline-rich receptor-like protein kinase PERK2
TF			
Unigene65053_xckb	U_D	F_F	MADS-box transcription factor 15
Unigene34602_xckb	U_F	F_F	Ethylene-responsive transcription factor ABI4
Unigene69673_xckb	U_F	F_F	Ethylene-responsive transcription factor ERF109
Resistance			
Unigene68271_xckb	U_D	F_F	Leucine-rich repeat extensin-like protein 3
CL2708.Contig2_xckb	U_D	F_F	Pollen-specific leucine-rich repeat extensin-like protein 1
Unigene72805_xckb	U_D	F_F	Resistance protein RGA2
CL6805.Contig1_xckb	F_U	F_F	Hydroxyproline-rich glycoprotein gp1
Unigene48841_xckb	F_U	F_F	Hydroxyproline-rich glycoprotein gp1
Unigene46836_xckb	U_D	F_F	Hydroxyproline-rich glycoprotein gp1
Unigene72060_xckb	U_D	F_F	Hydroxyproline-rich glycoprotein gp1

## Conclusions

This study, compared to identification of resistance genes against other plant parasitic nematodes, is the first to present large-scale identification of wheat resistance genes against cereal cyst nematode *H. avenae* using comparative transcriptomic analysis of incompatible and compatible reactions. In total, 606 candidate resistance genes including 302 primarily identified overlapping genes, 184 additional rescued genes, and 122 VP1620-specific genes, were identified on a large scale and functionally classified into diverse defense-related pathways. Phospholipases might play vital roles in early defense responses to *H. avenae* by involving diverse defense-related pathways as a hub switch. In addition, the plant-pathogen interaction pathway was significantly enriched and also played key roles in defense against *H. avenae*. These data not only provide applicable candidate resistance genes for breeding novel wheat cultivars but also strengthen new insights into defense responses to *H. avenae*, especially a better understanding of the mechanism underlying early defense response of cereal crops to cereal cyst nematode *H. avenae*.

## Materials and methods

### Nematode infection manipulation

Cereal cyst nematode *H. avenae* was maintained on a compatible wheat WEN19 at 16 °C in a greenhouse, mature cysts were collected, and pre-J2 were hatched as previously described [2, 8]. Both WEN19 and VP1620 were maintained at 16 °C in a greenhouse, and one-week-old wheat seedlings were inoculated with freshly hatched *H. avenae* as described previously [2]. To block continuous infection of *H. avenae*, the roots of both groups of infected plants were rinsed with double distilled water, and the rinsed plants were transferred to new pots for further growth [12]. The infected root samples of both WEN19 and VP1620 were harvested at 24 h, 3 d and 8 d, and the corresponding root samples without CCN inoculation were also collected as controls. Among these root samples, a subset were used to examine the number of different developmental CCN within the roots by staining the CCN with 0.01 % acid fuchsin solution as previously described [76, 77]; the others were subjected to RNA isolation with TRIzol reagent following the manufacturer’s instructions (Invitrogen).

### Bioinformatic analysis

Total RNAs were subjected to RNA-sequencing by Illumina Hiseq™ 2000. To remove CCN transcripts from raw transcriptomes, RNA-Seq data was mapped to CCN transcripts using SOAP [78], and unmapped reads from each sample were used for assembly in the next step. Transcriptome assemblies were performed with Trinity [79]. The RNA-Seq data from each sample of _I or _C was assembled together. Transcripts from _I and _C were used in a further process for redundancy removal using the TGI Clustering Tool (http://sourceforge.net/projects/tgicl/) and BLASTN. The acquired overlap and unique transcripts were used for further analyses. The BLASTX alignments (e-value < 0.00001) between transcripts and protein databases (NR, Swiss-Prot, KEGG and COG) were performed. The best hits were used to decide on sequence direction of transcripts. If hits of different databases conflicted with each other, a priority order of NR, SwissProt, KEGG and COG was followed for deciding the sequence direction of transcripts. For transcripts with no hits in these databases, ESTScan [80] was introduced to decide their sequence directions. To annotate functions of transcripts, they were searched against databases NT (BLASTN, e-value < 1e-5), NR, Swiss-Prot, KEGG and COG (BLASTX, e-value < 1e-5). The Blast2GO program [81] was used to obtain Gene Ontology annotation of transcripts, and metabolic pathways of transcripts were analyzed by Path_finder [82]. The expressions of genes were measured by Fragments Per kb per Million Fragments (FPKM) [83]. The algorithm used to identify differentially expressed genes was similar to that previously reported [84]. The calculated *p*-values were corrected using the Bonferroni method. A gene was considered to be differentially expressed between two samples if the corrected *p*-value (FDR) ≤ 0.001 and the fold change ≥ 2.

### Measurement of ROS level

The ROS levels within root samples were determined via luminol-chemiluminescence assay with minor modifications [85]. In a brief, root samples of both WEN19 and VP1620 collected at 24 h, 3 d and 8 d were cut and dropped into double distilled water for 4 h. An amount of 10 mg root per sample was placed in Eppendorf tubes containing 100 μL of luminal (Bio-Rad Immun-Star horseradish peroxidase substrate) and 1.0 μL of horseradish peroxidase (Jackson ImmunoResearch). Luminescence was immediately measured at 1 min intervals for 30 min with a Glomax 20/20 luminometer (Promega) [85]. Three replicates were performed for each sample and treatment. Standard errors were calculated for each treatment.

A total of 12 RNAs were treated with DNase to remove contaminating gDNA. First-strand cDNA was synthesized using the Invitrogen Superscript III Kit [52, 86], and qRT-PCR assay was performed using the iQ5 real-time PCR detection system (Bio-Rad). The actin gene was used as endogenous reference [87], and relative expression was determined as previously described [52]. Mean and standard errors were determined with data from three independent replicates. The primers used in this study are listed in Additional file 6: Table S4.

## Additional files

Additional file 1: Figure S1. VP1620 disturbed developmental transitions of *H. avenae. H. avenae* at developmental post-J2, J3 and J4 stages within VP1620 and WEN19 were examined by staining with acid fuchsin at 14 d, 19 d, 25 d, and 33 d. The developmental transitions within VP1620 were significantly affected compared to those in WEN19 (scale bar = 500 μm). (PPTX 540 kb) Additional file 2: Table S1. Number and percentage of *H. avenae* juveniles in wheat roots of both compatible and incompatible wheat lines at different time-course points. (DOCX 16 kb) Additional file 3: Figure S2. Identification of dramatically upregulated genes within VP1620 at three time points. Venn diagram showing the numbers of genes significantly upregulated at 24 h, 3 d and 8 d, respectively. Overlapping numbers indicate the amount of overlapping genes simultaneously and significantly upregulated among two or three time points. (PPTX 42 kb) Additional file 4: Table S2. Annotation of 18 transcripts with dramatically and simultaneously induction among three time points. (DOCX 17 kb) Additional file 5: Table S3. KEGG pathways with candidate resistance genes. (DOCX 21 kb) Additional file 6: Table S4. Identification of ROS-producing genes and primers used for qRT-PCR assay. (XLSX 11 kb) Additional file 7: Table S5. Additional rescued candidate resistance genes. (XLSX 11 kb) Additional file 8: Table S6. KEGG enrichment analysis of the rescued genes exhibiting uncoordinated expression profiles. (XLSX 16 kb) Additional file 9: Figure S3. Expression profiles of VP1620-specific genes. Cross-table representation of the expression profiles of VP1620-unique genes within the I_CN and I_0 groups. Numbers representing significantly upregulated genes induced by CCN infestation in each cluster combination within the dataset are indicated in each square. The top and left squares show trend lines for changes in expression pattern across three time points. Abbreviation: U, up-forward trend; D, down-forward trend; F, unchanged trend. ^a^ Total numbers of genes displaying expression profiles of each I_CN combined with nine I_0; ^b^ Total numbers of genes displaying similar expression profiles on the diagonal line; ^c^ Total numbers of genes displaying uncoordinated expression profiles outside the diagonal line. (PPTX 101 kb)

## Abbreviations

BR: Brassinosteroid
CCN: Cereal cyst nematode
CEBiP: Chitin elicitor-binding proteins
EFR: Elongation factor Tu (EF-Tu) receptor
FLS2: Flagellin sensitive2
LOX: Lipoxygenase
LRR: Leucine-rich repeat
JA: Jasmonic acid
MAPKs: Mitogen-acitivated protein kinases
NO: Nitric oxide
PAL: Phenylpropanoid ammonia-lyase
PAMPs: Pathogen-activitated molecular patterns
PLD: Phospholipase D
Post-J2: Post-parasitic Juvenile 2
POX: Class III peroxidase
PPI: Plant-pathogen interaction
PR: Pathogenesis-related
PTI: PAMP-trigered immunity
RLK: Receptor-like kinase
ROS: Reactive oxygen species
