# Supplementary figures and images for: Large-scale identification of wheat genes resistant to cereal cyst nematode Heterodera avenae using comparative transcriptomic analysis
Source: BMC Genomics. 2015 Oct 16;16:801. doi: 10.1186/s12864-015-2037-8 (PMC4609135; doi:10.1186/s12864-015-2037-8)

## Slide 1
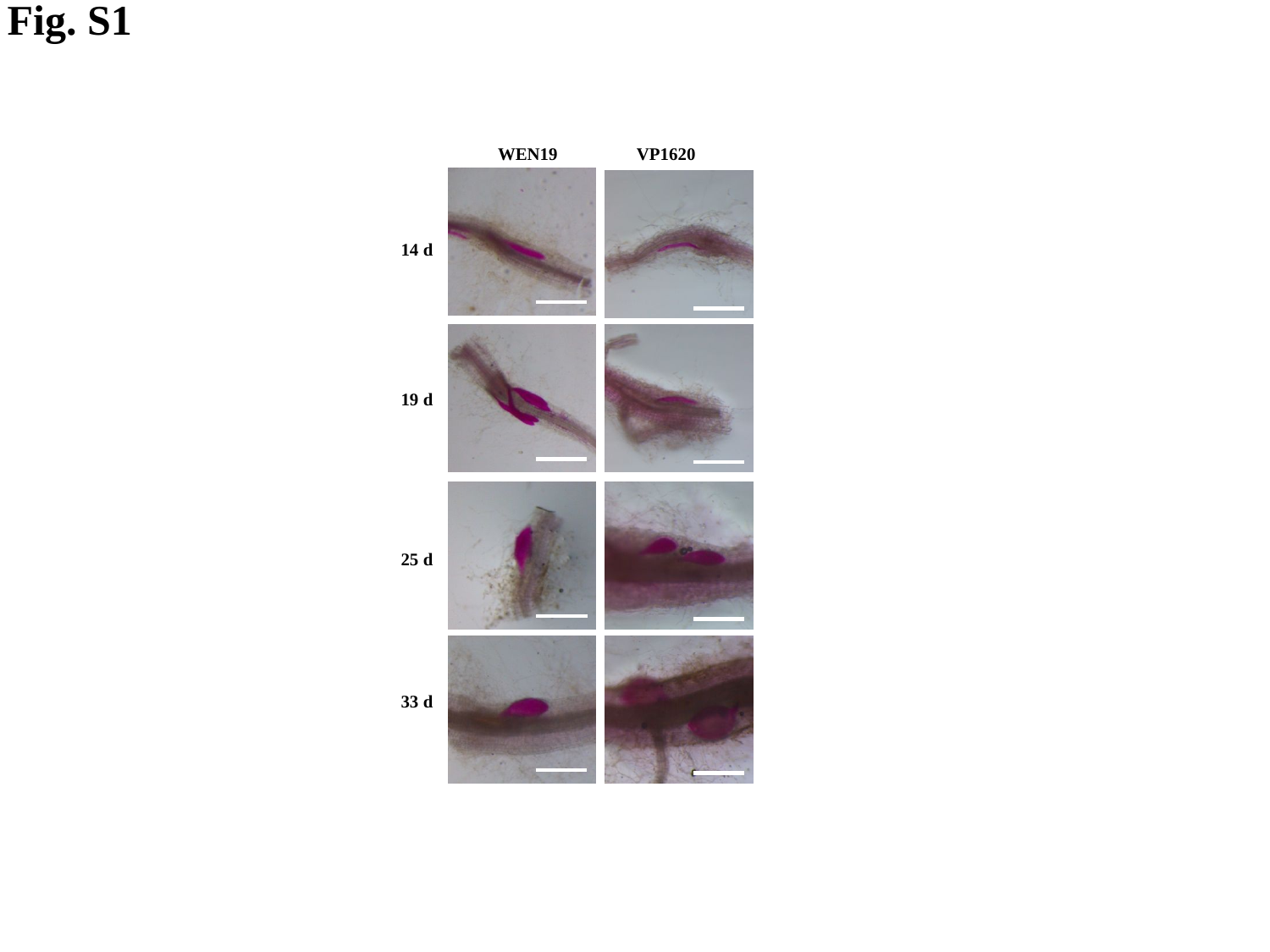

Fig. S1
WEN19
VP1620
14 d
19 d
25 d
33 d

Supplement: Additional file 1: Figure S1. — VP1620 disturbed developmental transitions of H. avenae. H. avenae at developmental post-J2, J3 and J4 stages within VP1620 and WEN19 were examined by staining with acid fuchsin at 14 d, 19 d, 25 d, and 33 d. The developmental transitions within VP1620 were significantly affected compared to those in WEN19 (scale bar = 500 μm). (PPTX 540 kb) [file 12864_2015_2037_MOESM1_ESM.pptx]

## Slide 1
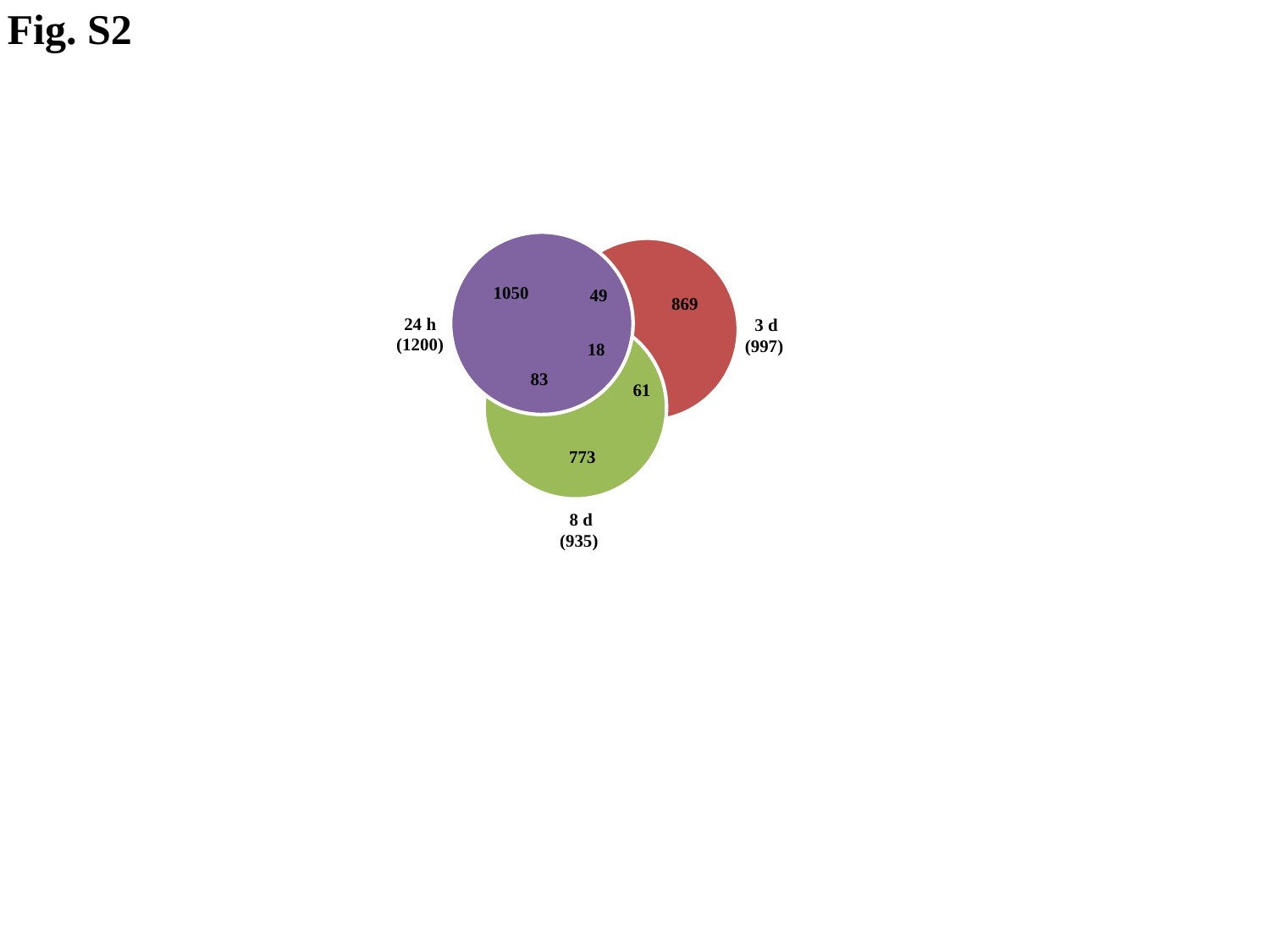

Fig. S2
1050
49
869
24 h
(1200)
 3 d
(997)
18
83
61
773
 8 d
(935)

Supplement: Additional file 3: Figure S2. — Identification of dramatically upregulated genes within VP1620 at three time points. Venn diagram showing the numbers of genes significantly upregulated at 24 h, 3 d and 8 d, respectively. Overlapping numbers indicate the amount of overlapping genes simultaneously and significantly upregulated among two or three time points. (PPTX 42 kb) [file 12864_2015_2037_MOESM3_ESM.pptx]

## Slide 1
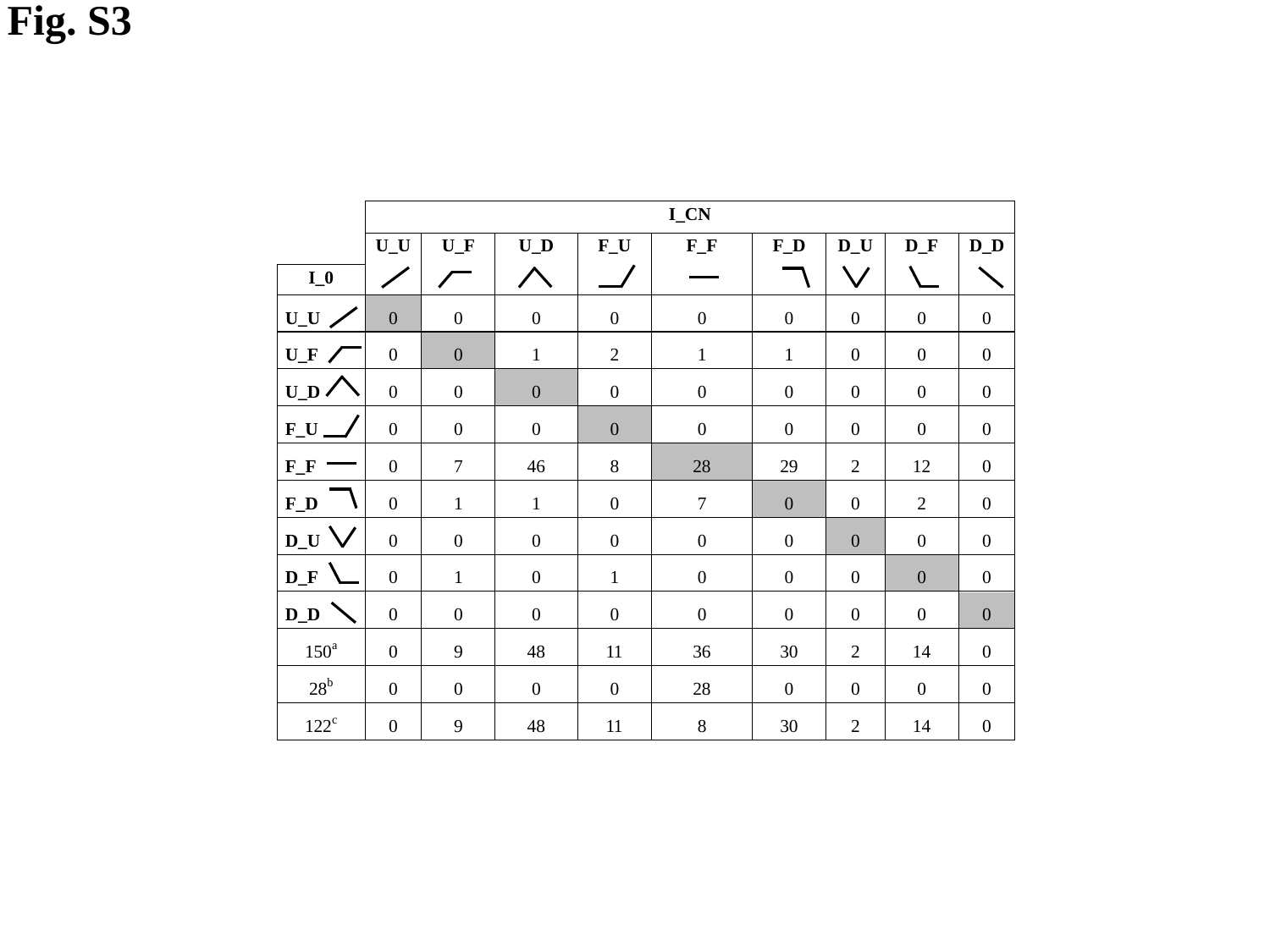

Fig. S3

Supplement: Additional file 9: Figure S3. — Expression profiles of VP1620-specific genes. Cross-table representation of the expression profiles of VP1620-unique genes within the I_CN and I_0 groups. Numbers representing significantly upregulated genes induced by CCN infestation in each cluster combination within the dataset are indicated in each square. The top and left squares show trend lines for changes in expression pattern across three time points. Abbreviation: U, up-forward trend; D, down-forward trend; F, unchanged trend. a Total numbers of genes displaying expression profiles of each I_CN combined with nine I_0; b Total numbers of genes displaying similar expression profiles on the diagonal line; c Total numbers of genes displaying uncoordinated expression profiles outside the diagonal line. (PPTX 101 kb) [file 12864_2015_2037_MOESM9_ESM.pptx]
