# Supplementary material for: Large-scale identification of wheat genes resistant to cereal cyst nematode Heterodera avenae using comparative transcriptomic analysis
Source: BMC Genomics. 2015 Oct 16;16:801. doi: 10.1186/s12864-015-2037-8 (PMC4609135; doi:10.1186/s12864-015-2037-8)
Supplement: Additional file 2: Table S1. — Number and percentage of H. avenae juveniles in wheat roots of both compatible and incompatible wheat lines at different time-course points. (DOCX 16 kb) [file 12864_2015_2037_MOESM2_ESM.docx]

**Table S1. Number and percentage of *H. avenae* juveniles in wheat roots of both compatible and incompatible wheat lines at different time-course points**

| **Time** | **Wheat line** | **No. of CCN**  **in roots** | **J2 (%)*** | **J3 (%)** | **J4 (%)** |
| --- | --- | --- | --- | --- | --- |
| 14 d | WEN19 | 54.3 ± 6.8^a^ | 47.5 ± 4.8 ^a^ | 52.5 ± 7.9 ^a^ | 0 |
|  | VP1620 | 42.7 ± 5.5^b^ | 56.2 ± 7.0 ^b^ | 43.8 ± 5.9 ^b^ | 0 |
| 19 d | WEN19 | 39.7 ± 5.9 ^a^ | 6.2 ± 5.2 ^a^ | 93.8 ± 15.1 ^a^ | 0 |
|  | VP1620 | 30.3 ± 1.8 ^b^ | 48.0 ± 3.8 ^b^ | 52.0 ± 1.5 ^b^ | 0 |
| 25 d | WEN19 | 38.3 ± 2.5 ^a^ | 0 ^a^ | 96.6 ± 8.1 ^a^ | 3.4 ± 3.0 ^a^ |
|  | VP1620 | 19.3 ± 3.7 ^b^ | 12.6 ± 5.6 ^b^ | 87.4 ± 16.6 ^b^ | 0 ^b^ |
| 33 d | WEN19 | 14.3 ± 2.2 ^a^ | 0 | 24.3 ± 9.1 ^a^ | 75.7 ± 6.7 ^a^ |
|  | VP1620 | 8.3 ± 1.6 ^b^ | 0 | 88.0 ± 6.9 ^b^ | 12.0 ± 12.0 ^b^ |

* Represents the percentage of each stage of CCN in each time point. Different letters above the number denote significant differences (P value ≤0.05, n=3)
