# Supplementary material for: Large-scale identification of wheat genes resistant to cereal cyst nematode Heterodera avenae using comparative transcriptomic analysis
Source: BMC Genomics. 2015 Oct 16;16:801. doi: 10.1186/s12864-015-2037-8 (PMC4609135; doi:10.1186/s12864-015-2037-8)
Supplement: Additional file 4: Table S2. — Annotation of 18 transcripts with dramatically and simultaneously induction among three time points. (DOCX 17 kb) [file 12864_2015_2037_MOESM4_ESM.docx]

**Table S2. Annotation of 18 transcripts with dramatically and simultaneously induction among three time points**

| **Gene ID** | **24 h^*^** | **3d^**^** | **8d^***^** | **Nr-annotation** |
| --- | --- | --- | --- | --- |
| CL14562.Contig3_All | 4.3883 | 1.6827 | 1.8539 | Pathogenesis-related protein |
| Unigene36809_All | 9.8237 | 11.8598 | 9.6968 | Elongation factor-1 α-like protein |
| Unigene627_All | 11.3832 | 9.7139 | 11.3218 | Probable mediator of RNA polymerase II transcription subunit 37c |
| Unigene19712_All | 5.9502 | 2.9815 | 1.8758 | Cell wall-associated hydrolase |
| CL809.Contig1_All | 5.2013 | 2.9958 | 2.2247 | Cell wall-associated hydrolase, partial associated hydrolase |
| Unigene40922_All | 5.3494 | 2.9859 | 1.7654 | Cell wall-associated hydrolase, partial associated hydrolase |
| Unigene41047_All | 13.1755 | 10.5289 | 10.2657 | Curcuminoid synthase-like |
| Unigene40178_All | 14.3845 | 12.2794 | 5.021 | Predicted curcuminoid synthase-like |
| Unigene30868_All | 3.9591 | 2.3849 | 4.2889 | Predicted premnaspirodiene oxygenase-like |
| CL2952.Contig3_All | 1.3252 | 1.4317 | 1.7262 | Cytochrome P450 734A6 |
| Unigene48447_All | 1.6703 | 1.5232 | 2.6084 | Mitochondrial protein |
| Unigene23333_All | 3.0158 | 1.1544 | 1.3105 | Cationic peroxidase SPC4 |
| CL331.Contig1_All | 7.0543 | 3.7245 | 3.0069 | Cinnamoyl-CoA reductase 1 |
| CL3559.Contig1_All | 3.7187 | 1.1003 | 1.6153 | Probable apyrase 3 |
| CL14542.Contig1_All | 3.7192 | 4.3615 | 3.2658 | Hypothetical protein |
| Unigene15642_All | 8.7306 | 6.2554 | 4.9963 | Hypothetical protein |
| CL14331.Contig2_All | 6.4694 | 3.537 | 2.7217 | Hypothetical protein |
| CL14543.Contig1_All | 3.7771 | 1.1229 | 2.3846 | Hypothetical protein |

* log2 (I-24h_CN/I-24h_0);

** log2 (I-3d_CN/I-3d_0);

*** log2 (I-8d_CN/I-8d_0)
