# Supplementary material for: Large-scale identification of wheat genes resistant to cereal cyst nematode Heterodera avenae using comparative transcriptomic analysis
Source: BMC Genomics. 2015 Oct 16;16:801. doi: 10.1186/s12864-015-2037-8 (PMC4609135; doi:10.1186/s12864-015-2037-8)
Supplement: Additional file 5: Table S3. — KEGG pathways with candidate resistance genes. (DOCX 21 kb) [file 12864_2015_2037_MOESM5_ESM.docx]

**Table S3 KEGG pathways with candidate resistance genes**

| **KEGG pathway** | **24h_vs_3d*** | **3d_vs_8d**** | **Total***** |
| --- | --- | --- | --- |
| Endocytosis [PATH:ko04144] | 15 | 6 | 21 |
| RNA transport [PATH:ko03013] | 14 | 7 | 21 |
| Glycerophospholipid metabolism [PATH:ko00564] | 16 | 5 | 21 |
| cAMP signaling pathway [PATH:ko04024] | 15 | 5 | 20 |
| Ras signaling pathway [PATH:ko04014] | 15 | 5 | 20 |
| Ether lipid metabolism [PATH:ko00565] | 15 | 5 | 20 |
| Plant-pathogen interaction [PATH:ko04626] | 18 | 2 | 20 |
| mRNA surveillance pathway [PATH:ko03015] | 12 | 7 | 19 |
| DNA repair and recombination [PATH:ko03400] | 7 | 1 | 8 |
| Starch and sucrose metabolism [PATH:ko00500] | 7 | 1 | 8 |
| Pentose and glucuronate interconversions [PATH:ko00040] | 6 | 1 | 7 |
| Plant hormone signal transduction [PATH:ko04075] | 3 | 1 | 4 |
| RNA polymerase [PATH:ko03020] | 3 | 1 | 4 |
| Purine metabolism [PATH:ko00230] | 3 | 1 | 4 |
| Pyrimidine metabolism [PATH:ko00240] | 3 | 1 | 4 |
| Lysosome [PATH:ko04142] | 3 | 0 | 3 |
| Phagosome [PATH:ko04145] | 3 | 0 | 3 |
| Spliceosome [PATH:ko03040] | 2 | 1 | 3 |
| ABC transporters [PATH:ko02010] | 2 | 0 | 2 |
| Protein processing in endoplasmic reticulum [PATH:ko04141] | 0 | 2 | 2 |
| RNA degradation [PATH:ko03018] | 2 | 0 | 2 |
| Cysteine and methionine metabolism [PATH:ko00270] | 2 | 0 | 2 |
| Tryptophan metabolism [PATH:ko00380] | 0 | 2 | 2 |
| Benzoxazinoid biosynthesis [PATH:ko00402] | 1 | 1 | 2 |
| Biosynthesis of amino acids [PATH:ko01230] | 2 | 0 | 2 |
| Cell cycle [PATH:ko04110] | 1 | 0 | 1 |
| p53 signaling pathway [PATH:ko04115] | 1 | 0 | 1 |
| Gap junction [PATH:ko04540] | 1 | 0 | 1 |
| Regulation of autophagy [PATH:ko04140] | 1 | 0 | 1 |
| AMPK signaling pathway [PATH:ko04152] | 1 | 0 | 1 |
| Calcium signaling pathway [PATH:ko04020] | 1 | 0 | 1 |
| cGMP - PKG signaling pathway [PATH:ko04022] | 1 | 0 | 1 |
| SNARE interactions in vesicular transport [PATH:ko04130] | 1 | 0 | 1 |
| Homologous recombination [PATH:ko03440] | 1 | 0 | 1 |
| Basal transcription factors [PATH:ko03022] | 1 | 0 | 1 |
| Aminoacyl-tRNA biosynthesis [PATH:ko00970] | 0 | 1 | 1 |
| Phenylalanine metabolism [PATH:ko00360] | 1 | 0 | 1 |
| Glucosinolate biosynthesis [PATH:ko00966] | 0 | 1 | 1 |
| Phenylpropanoid biosynthesis [PATH:ko00940] | 1 | 0 | 1 |
| Fructose and mannose metabolism [PATH:ko00051] | 1 | 0 | 1 |
| Galactose metabolism [PATH:ko00052] | 1 | 0 | 1 |
| Glycolysis / Gluconeogenesis [PATH:ko00010] | 1 | 0 | 1 |
| Pentose phosphate pathway [PATH:ko00030] | 1 | 0 | 1 |
| N-Glycan biosynthesis [PATH:ko00510] | 1 | 0 | 1 |
| Various types of N-glycan biosynthesis [PATH:ko00513] | 1 | 0 | 1 |
| Cutin, suberine and wax biosynthesis [PATH:ko00073] | 1 | 0 | 1 |
| Carotenoid biosynthesis [PATH:ko00906] | 1 | 0 | 1 |
| 2-Oxocarboxylic acid metabolism [PATH:ko01210] | 0 | 1 | 1 |
| Carbon metabolism [PATH:ko01200] | 1 | 0 | 1 |
| Circadian rhythm - plant [PATH:ko04712] | 1 | 0 | 1 |

*Number of candidate resistance genes exhibiting increasing expression trends in the 24h_vs_3d stage enriched in the KEGG pathways; **Number of candidate resistance genes exhibiting increasing expression trends in the 3d_vs_8d stage enriched in the KEGG pathways; ***Total number of candidate resistance genes exhibiting increasing expression trends in both 24h_vs_3d and 3d_vs_8d stage enriched in the KEGG pathways.
